# Towards a wavefront-preservation X-ray crystal monochromator for high-repetition-rate FELs

**DOI:** 10.1107/S1600577523004216

**Published:** 2023-06-15

**Authors:** Lin Zhang, Matthew Seaberg, Hasan Yavaş

**Affiliations:** a Linac Coherent Light Source, SLAC National Accelerator Laboratory, 2575 Sand Hill Road, Menlo Park, CA 94025, USA; Uppsala University, Sweden

**Keywords:** X-ray optics, monochromator crystal, thermal deformation, wavefront preservation, Strehl ratio, FEL

## Abstract

The wavefront preservation of coherent X-ray free-electron laser beams is pushing the requirement on the quality and performance of X-ray optics to an unprecedented level. The Strehl ratio can be used to quantify this requirement. In this paper, the criteria for thermal deformation of the X-ray optics are formulated, especially for crystal monochromators. To preserve the X-ray wavefront, the standard deviation of the height error should be sub-nm for mirrors and less than 25 pm for crystal monochromators. Cryocooled silicon crystals combined with two techniques can be used to achieve this level of performance for monochromator crystals: (1) using a focusing element to compensate the second-order component of the thermal deformation; (2) introducing a cooling pad between the cooling block and silicon crystal and optimizing the effective cooling temperature. Each of these techniques allows the thermal deformation in standard deviation of the height error to be reduced by an order of magnitude. As an example, for the LCLS-II-HE Dynamic X-ray Scattering instrument, the criteria on thermal deformation of a high-heat-load monochromator crystal can be achieved for a 100 W SASE FEL beam. Wavefront propagation simulations confirm that the reflected beam intensity profile is satisfactory on both the peak power density and focused beam size.

## Abstract 2

The thermal deformation requirement for wavefront preservation through an X-ray crystal monochromator is found to restrict the standard deviation of the height error to less than 25 pm under certain conditions. By optimizing the effective cooling temperature of liquid-nitro­gen-cooled crystals, combined with compensation of the second-order component of the thermal deformation, an approach to reach this unprecedented requirement is described.

## 1. Introduction

In the past decade, numerous coherent X-ray light sources such as high-repetition-rate X-ray free-electron lasers (XFELs) and diffraction-limited storage rings (DLSRs) based on multi-bend achromat (MBA) designs have been emerging around the world. The peak XFEL power has reached terawatt levels. The average power from high-repetition-rate XFELs can reach a few hundred watts. The requirement to preserve the wavefront of coherent X-ray beams leads to new engineering challenges in X-ray optics. Minimizing thermal deformation of X-ray optics with tens or hundreds of watts of power load demands increasingly innovative approaches.

With the advent of third-generation synchrotron light sources back to the late 1980s and 1990s, the X-ray power load on the optics reached several hundred watts. Thermal deformation of the optics can lead to photon flux reduction, divergent X-ray beams, and ultimately a significant reduction of the X-ray beam brilliance. Many engineering efforts and initiatives have been conducted across the world to minimize or compensate the thermal deformation of the X-ray optics. To quantify the thermal deformation, *i.e.* the shape variation due to the heat load, the thermal slope error Δθ or height error Δ*h* over the beam footprint on the optics are often used. The thermal slope error for constant material properties can be expressed as (Zhang *et al.*, 2014 ▸; Zhang, 2018 ▸)

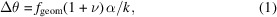

where α, *k* and ν are the thermal expansion coefficient, thermal conductivity and Poisson’s ratio of the substrate material, respectively, *f*_geom_ is a geometric function depending on the optics and cooling geometry, and the power distribution. There are two ways to minimize the thermal deformation: (1) minimizing the geometrical function *f*_geom_ by working on the cooling design and geometrical shape of the optics, and (2) minimizing the figure of merit in material properties (1 + ν)α/*k* by choosing the materials and/or the temperature range when the material properties are strongly temperature dependent. The current state-of-the-art solutions to minimize the thermal deformation of the X-ray optics include:

(1) Synchrotron X-ray white-beam mirrors to minimize the geometrical function *f*_geom_. Cool the mirror using water along the top sides of the substrate, fully illuminate (overfill) the mirror length, optimize the mirror cross section with notches, use a secondary slit downstream of the mirror to shape the beam to the desired final size (Zhang, 2010 ▸; Zhang *et al.*, 2013*a* ▸). The thermal slope error of such a mirror, with about 800 W of absorbed beam power, can be minimized to 0.018 µrad. This technique is widely used for white-beam mirrors and most white- or pink-beam multilayer optics at the ESRF Upgrade beamlines and APS-U beamlines (X. B. Shi, private communication). In comparison, RMS thermal slope errors in the case of mirror cooling on the bottom face (opposite the mirror optical surface) can reach 200 µrad. The concept of a mirror cross section with notch structures was also proposed previously (Khounsary, 1999 ▸).

(2) Cryo- or liquid-nitro­gen (LN2) cooling of the silicon crystal monochromator. The figure of merit of material properties, the ratio α/*k* of the silicon at LN2 temperature (77 K at 1 atm), is about 1–2% of that at room temperature. Therefore, LN2 cooling can significantly reduce the thermal deformation of the silicon crystal, compared with water cooling at near room temperature. LN2-cooled silicon crystal monochromators have been widely investigated (Marot *et al.*, 1992 ▸; Zhang, 1993 ▸; Rogers *et al.*, 1995 ▸; Lee *et al.*, 1995 ▸; Bilderback *et al.*, 2000 ▸; Lee *et al.*, 2000 ▸, 2001 ▸; Mochizuki *et al.*, 2001 ▸; Zhang *et al.*, 2003 ▸, 2013*b* ▸) and subsequently used in many beamlines.

(3) Actively compensated X-ray mirrors (flat or bendable): piezo-actuator dynamic bendable mirror (Susini *et al.*, 1992 ▸) or (multi-electrode) piezo bimorph mirror (Susini *et al.*, 1995 ▸). These active optics can either be bent for variable focusing or adaptively bent to compensate thermal deformation. For wavefront-preservation X-ray mirrors at Linac Coherent Light Source (LCLS), an active cooling and heating technique was proposed (Zhang *et al.*, 2015 ▸) and tested (Cocco *et al.*, 2020 ▸). This is a novel active/adaptive water-cooling technique – called REAL (resistive element adjustable length) cooling with applied auxiliary heating, tailored to the spatial distribution of the thermal load generated by the incident beam. Using this technique, we can theoretically achieve sub-nanometre surface figure errors on X-ray mirrors with up to several hundred watts of average incident beam power, for use with high-repetition-rate XFELs such as LCLS-II and LCLS-II-HE.

The LCLS-II-HE project includes doubling the electron energy to 8 GeV from the superconducting LINAC, and extending the photon energy range up to 18 keV and possibly to 25 keV. Crystal optics will be used for monochromators, delay lines and spectrometers due to the natural match between their interatomic distances and the hard X-ray wavelengths. Coherent high-average-power hard X-rays have recently become available at the European XFEL and soon at LCLS-II-HE, making wavefront preservation through crystal monochromators timely. This paper focuses on the thermal-mechanical requirements of such optics. We will describe how to translate these requirements to opto-mechanical engineering design criteria and performance measures. We will present a thermal-mechanical optimization method and application to the high-heat-load crystal monochromator (HHLM) of the LCLS-II-HE Dynamic X-ray Scattering (DXS) instrument. Wavefront simulation results of such monochromator crystals will also be reported and discussed.

## 2. Optics requirement for wavefront preservation

The performance requirements of X-ray optics since the advent of third-generation synchrotron light sources are mostly related to the preservation and improvement of the quality of the photon beam, such as the photon flux, beam collimation, beam focusing, harmonic rejection, monochromatization and beam stability. Wavefront preservation becomes an increasing requirement on the X-ray optics for coherent high-power X-ray light sources. It is useful at this stage to define a quantitative measure of this requirement on the optics. One approach is to compare the X-ray beam intensity in the focal plane after reflection from an aberrated optical system with the perfect case. The ratio of the real versus ideal peak intensity is widely known as the Strehl ratio (Strehl, 1895 ▸, 1902 ▸). When the quality of the optics is close to perfect, the Strehl ratio tends to 1. The Strehl ratio (SR) can also be calculated based on the root mean square of the phase error (φ) introduced by the non-perfect optics, and can be written as

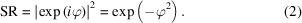

**Figure 1:**
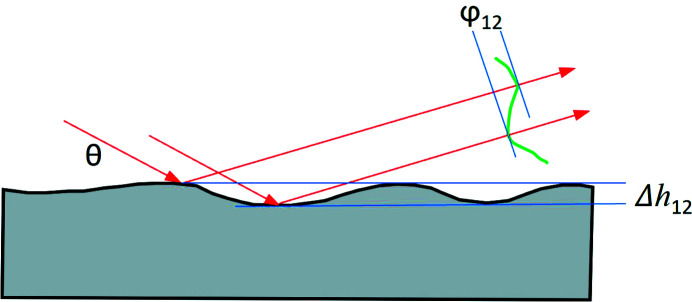
Schematic of an X-ray mirror with surface shape height error Δ*h* and consequence on phase error φ of the X-ray beam with incidence angle θ.

The optical path length difference Δ*L*_12_ between the two rays with glancing incident angle θ reflected by an X-ray mirror with a surface shape height error Δ*h*_12_ (Fig. 1 ▸) can be calculated as Δ*L*_12_ = 2Δ*h*_12_sin(θ). The phase error is then φ_12_ = 2πΔ*h*_12_/λ where λ is the beam wavelength. Now let us assume that Δ*h* is the standard deviation (or RMS value with the reference at average height) of the height error over the beam footprint on the optic, and φ is the RMS phase error over the cross section of the reflected beam. This phase error is given by

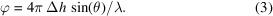

Here, the units of Δ*h* and λ are the same, for instance nm; the units of phase error φ are radians. In the literature, some authors omitted 2π or a factor of 2.

For a given Strehl ratio requirement, we can deduce the requirement in shape height error Δ*h* of the X-ray optics. This requirement on the height error can be used as criteria for heat-load-induced thermal deformation, as well as polishing, manufacturing and mounting errors of the optics.

To minimize the thermal deformation of an X-ray mirror subjected to a photon beam with incidence angle θ and photon energy *e*_ph_ (keV), we can use the following criterion (Zhang *et al.*, 2015 ▸),

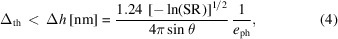

where Δ_th_ is the RMS height error (in nm) induced by the photon beam heat load.

**Figure 2:**
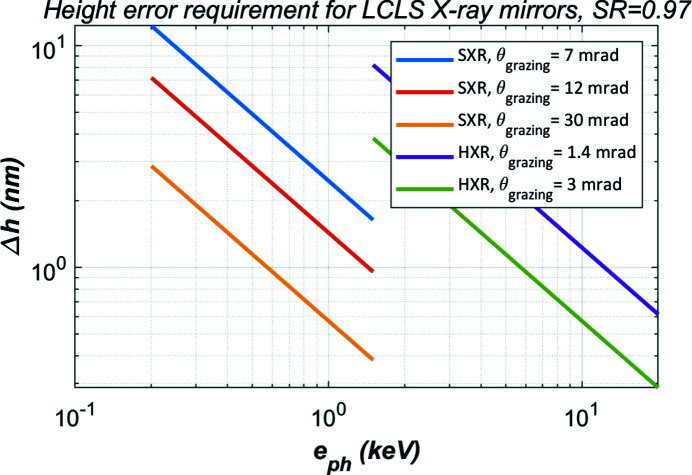
Height error requirement for LCLS X-ray mirrors at different beam grazing incidence angle θ.

For the LCLS-II soft X-ray (SXR) mirrors, incidence angles are between 7 and 30 mrad. For the LCLS-II-HE hard X-ray (HXR) mirrors, grazing incidence angles are between 1.4 and 3 mrad. The RMS height error requirements Δ*h* for the LCLS-II X-ray mirrors are plotted in Fig. 2 ▸. For higher Strehl ratio requirements, the threshold value Δ*h* should be smaller. As the mirror grazing incidence angle is small, sinθ ≅ θ, and the threshold value Δ*h* is inversely proportional to the grazing angle θ and photon energy *e*_ph_. The effects of smaller grazing incidence angle of LCLS HXR mirrors compared with SXR mirrors on the Δ*h* requirement quite offset the effects of higher photon energy with HXR mirrors. The requirements on height error are similar for the SXR and HXR mirrors. The thermal deformation of the X-ray mirrors should be limited to sub-nanometres in RMS height error.

For crystal monochromators, the deformation of the crystal plane should be considered rather than the surface. We use the term ‘height error’ mostly to differentiate from the ‘slope error’ for crystal monochromators, but both refer to the deformation of the crystal plane. The same criterion as for the mirror in equation (3) can be used to minimize the effects of thermal deformation. The beam incident angle (θ), wavelength (λ) or photon energy (*e*_ph_), and crystal *d*-spacing parameter *d* are governed by Bragg’s law,

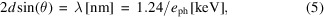

where the units of *d* are nm. For a crystal diffraction plane (*ijk*), the *d*-spacing parameter is *d*_*ijk*_ = *a*/(*i* ^2^ + *j* ^2^ + *k* ^2^)^1/2^, where *a* is the lattice constant (*a* = 0.543 nm for silicon crystal). Note that the crystal monochromator works for HXR, for instance above 2 keV with a silicon crystal. Combining equations (2), (3) and (5), one obtains the following,

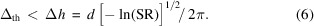

**Figure 3:**
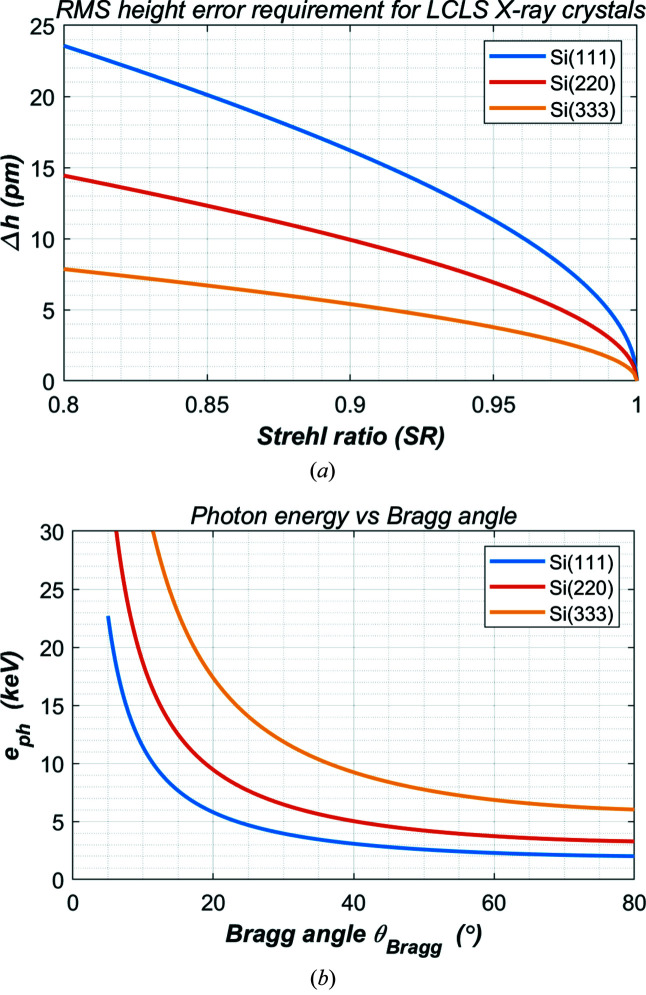
For LCLS X-ray monochromator crystals, (*a*) the requirement in height error of crystal planes versus Strehl ratio, and (*b*) the photon energy range versus Bragg angle when using the three listed crystals.

The relation [equation (3)] between the threshold value Δ*h* and Strehl ratio for a crystal is the same as for a mirror. But the criterion to limit the thermal deformation or other aberration on the crystal monochromator depends only on the crystal *d*-spacing parameters. The influences of the beam incident angle and photon energy on this requirement are indirect through the *d*-spacing parameter as equation (5). The threshold Δ*h* versus Strehl ratio for various silicon crystals is shown in Fig. 3 ▸(*a*). To reach a Strehl ratio of higher than 0.8, the distortion of the crystal planes should be limited to smaller than 25 pm in RMS height error for Si(111), and smaller than 10 pm for Si(333). These numbers are about 50 times smaller than the threshold for mirrors. This big difference in requirement between mirror and monochromator crystal is basically related to the beam incident angle. For example, the grazing incident angles for an HXR mirror are about 2 mrad; Bragg angles for the three silicon crystals shown in Fig. 3 ▸(*b*) in the photon energy range up to 20 keV are larger than 100 mrad. The beam incident angle of the silicon crystal is about 50 times that of the grazing incident angle of the HXR mirrors.

In latter sections, we will show that the 10–25 pm height error requirements for crystal planes are relevant and appropriate for the coherent wavefront preservation of the X-ray beam. The Strehl ratio can be calculated from the thermal deformation height error as for monochromator crystals,

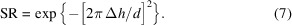

Here the units of both Δ*h* and *d* are nm. For mirrors,

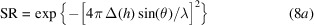

or

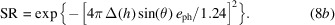

Here the units of both Δ*h* and λ are nm and those of photon energy *e*_ph_ are keV.

## 3. Optics requirement for wavefront preservation

The power load on the X-ray optics for the new coherent X-ray light sources (high-repetition-rate FEL and MBA lattice DLSR) reaches a few hundred watts. Minimizing thermal deformation of the X-ray optics becomes critical for the wavefront preservation that is required for coherent beams.

We have mentioned some of the state-of-the-art solutions to minimize the thermal deformation of the X-ray optics. For the crystal monochromator, cryo-cooled silicon crystals have been widely used for the third-generation synchrotron light sources. LN2 is the typical coolant used due to its high cooling capacity and better stability compared with pulse tube cryocoolers.

The X-ray beam power-load-induced temperature gradient is the driving force for the thermal deformation of the crystal. The thermal mechanical properties of silicon, such as thermal conductivity and thermal expansion coefficient, are temperature dependent. The thermal expansion coefficient of silicon is zero around 125 K. The optimum temperature level of the crystal should be such that the temperature in the beam footprint adjacent volume is around 125 K; the maximum temperature should be higher than 125 K. The objective function of the optimization is the thermal deformation, for instance the standard deviation of the height error over the beam footprint.

**Figure 4:**
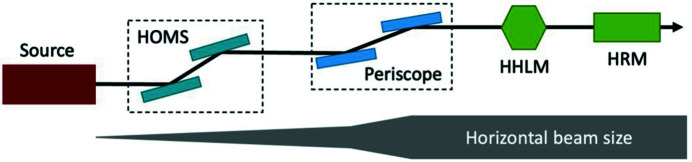
DXS optical layout (top view). At the very basic level, the DXS optical layout is composed of two pairs of mirror systems – a hard X-ray offset mirror system (HOMS) and periscope mirrors – and two monochromator systems (HHLM and HRM). The X-ray beam is enlarged and collimated by the periscope mirrors to enhance optical performance.

In this paper, we will present the thermal deformation minimization of the crystal monochromators for the LCLS-II-HE DXS instrument (or beamline as it is called in the synchrotron radiation community). The key optics components in the DXS instrument (layout shown in Fig. 4 ▸) include (1) a first pair of horizontal deflecting offset mirrors for hard X-ray beam transport, (2) a pair of dynamically bendable defocusing/focusing mirrors – called ‘periscope mirrors’; (3) a high-heat-load monochromator (HHLM); and (4) 4f high-resolution monochromator (4f-HRM). The maximum beam power load is 100 W which could be from a SASE or seeded FEL beam. To focus on the methodology of the optimization, we consider the first crystal in the HHLM, which absorbs the most power load among all the crystals used in DXS monochromators. This first crystal can absorb up to two-thirds of the total beam power from a SASE FEL. The photon energy range of the DXS instrument is 6–25 keV.

The design and optimization of the DXS HHLM is ongoing. In this paper, we will focus on the conceptual design and methodology to minimize the thermal deformation of the crystal monochromator with the objective to satisfy the design criteria shown in Section 2. A detailed design of the HHLM assembly is not the subject of this paper.

### 3.1. Finite-element models (FEMs)

**Figure 5:**
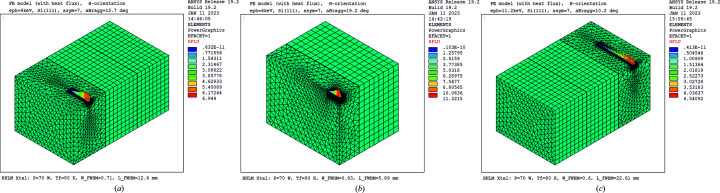
Finite-element model of the first crystal block with Gaussian power distribution loading (units: W mm^−2^). Meshing of the crystal with power load is identical whenever the centre crystal C_1-1_ or side crystals C_1-2_, C_1-3_ were used. (*a*) C_1-2_ (α_asym_ = 7, e_ph_ = 9 keV). (*b*) C_1-1_ (α_asym_ = 7, e_ph_ = 6 keV). (*c*) C_1-3_ (α_asym_ = 7, e_ph_ = 11.2 keV).

At one stage of the design optimization of the DXS HHLM, a two double-crystal monochromator (2×DCM) scheme was considered. The first crystal block in the first DCM was composed of three crystals side-by-side with different crystal planes and different asymmetry cut angles. Each of the crystals in this first crystal block is a rectangular shape of dimensions 75 mm in length, 20 mm in width and 30 mm in thickness. Any one of these three crystals could be used by lateral translation of the crystal block. A 0.2 mm-thick indium foil was assumed to be used between the crystals, as well as between the crystal and cooling block. In this study, finite-element modelling is applied only to the silicon crystal block shown in Fig. 5 ▸, as in previous studies (Zhang, 1993 ▸; Zhang *et al.*, 2003 ▸, 2013*b* ▸), and to the 0.2 mm-thick indium foil between crystals. The crystal block was cooled on the two external side areas with an effective cooling coefficient of *h*_cv_ and effective cooling temperature of *T*_cool_. A value of *h*_cv_ = 3000 W m^−2^ K^−1^ was used in the simulation. The effective thermal conductance between two crystals was 0.005 W m^−2^ K^−1^. These numbers are consistent with the data reported by Zhang *et al.* (2013*b* ▸) and Lee *et al.* (2020 ▸). Gaussian power distribution and surface power loading were used in these simulations. The Gaussian parameters were calculated using the corresponding Bragg angle, and results of an LCLS-II-HE Start-to-End (S2E) simulation on photon beam size and divergence versus photon energy. As examples, Fig. 5 ▸ shows the FEM with power load distribution. Details of the corresponding photon energy, asymmetry cut angle and meshing of the crystals are indicated in the figure caption.

### 3.2. Thermal deformation minimization by optimizing cooling temperature

The proposed crystal size in Section 3.1 was based on preliminary optimization simulations. At those crystal dimensions, the thermal deformation of the crystal does not vary with crystal size. Thermal deformation of the crystal depends on the total absorbed power, on the power distribution (Gaussian parameters) and Bragg angle – both of which depend on the photon energy. As mentioned previously, the thermal deformation of a cryo-cooled crystal can be minimized by controlling the temperature of the crystal in the beam footprint adjacent volume at around 125 K. The effective cooling coefficient *h*_cv_ is not a convenient controllable parameter. Once the cooling technique is chosen, and the geometry of the cooling block and the interface with the crystal defined, the cooling coefficient *h*_cv_ is mostly fixed. The effective cooling temperature *T*_cool_ can be a controllable parameter. This can be realized by using a pulsed cryocooler cold head, or by an LN2 cryocooler combined with electric heater strategically placed on an intermediate cooling pad between the crystal blocks and cooling pipes, as illustrated for the LCLS REAL cooled mirror (Zhang *et al.*, 2015 ▸). In the case of LN2 cooling, *T*_cool_ should be above 77 K.

We can optimize the cooling temperature by minimizing the thermal deformation of the crystal for the whole photon energy range 6–25 keV. But we will focus on several photon energies at which most of the science cases are anticipated. Thermal deformation of the crystal can be quantified by the standard deviation of the height error which is the thermal deformation displacement (*Uy*) of the crystal surface over the footprint length, denoted std_*Uy*_. With dynamic focusing elements (periscope mirrors) on the instrument (beamline), we can compensate the second-order component in the thermal deformation. Denoting the best spherical shape fit of the thermal deformation displacement as *Uy-fit*, the residual height error is then d*Uy* = *Uy* − *Uy-fit*. The standard deviation of the residual height error is noted as std_d*Uy*_. Both height error std_*Uy*_ or residual height error std_d*Uy*_ can be used as the objective function for thermal deformation minimization.

The periscope mirrors and 4f-HRM all operate on the horizontal reflection/diffraction plane. For 4f-HRM, horizontal geometry is essential since the hard X-ray beam of LCLS is vertically polarized. Therefore, wavefront preservation in the horizontal direction is crucial. HHLM crystals can be either in the vertical or horizontal diffraction scheme since the Bragg angles tend to be smaller, and so are the resulting polarization effects. We will simulate these two cases and compare them to finally decide which orientation will be chosen. For both cases, we will focus on the thermal deformation of the crystal in the horizontal direction. This will be the meridional direction for the horizontal oriented crystal or sagittal (or transverse) direction for the vertical oriented crystal.

### 3.3. FEA results

**Figure 6:**
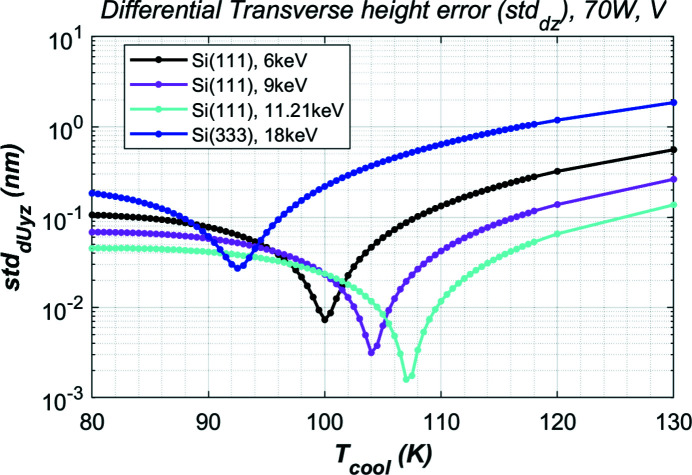
Residual height error of the crystal in the transverse direction (or horizontal for the photon beam) calculated with different effective cooling temperatures *T*_cool_ for the four cases listed in Table 1 ▸ and 70 W absorbed power.

The HHLM at the DXS instrument will cover a photon energy range between 6 and 18 keV. For a vertical oriented crystal and 70 W absorbed power (more than 100 W of FEL power from the source), the residual height error of the crystal in the transverse direction (or horizontal for the photon beam) calculated with different effective cooling temperature *T*_cool_ is shown in Fig. 6 ▸ for the four typical photon energies: 6, 9, 11.21, 18 keV. For each photon energy case, there is an optimal *T*_cool_ at which the residual height error std_d*Uy*_ is minimum. By optimizing the cooling temperature, the residual height error can be reduced by about a factor of ten compared with standard LN2 cooling where the effective cooling temperature *T*_cool_ is about 80 K. This optimal cooling temperature depends on the photon energy and crystal Bragg and asymmetry cut angles. The values of this optimal cooling temperature and the maximum temperature in the crystal are given in Table 1 ▸. In all cases the maximum temperature is above 125 K. We can see the trend that, when the maximum temperature of the crystal closer to 125 K or the temperature difference between the maximum temperature of the crystal and the cooling temperature is smaller, the thermal deformation is smaller.

**Figure 7:**
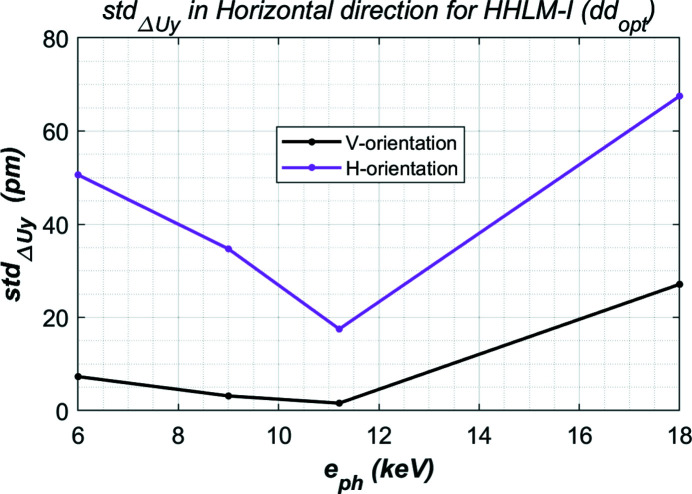
Minimized residual height error std_d*Uy*_ in the (beam) horizontal direction for both the vertical and horizontal orientated crystal for the four cases listed in Table 1 ▸ and 70 W absorbed power.

**Figure 8:**
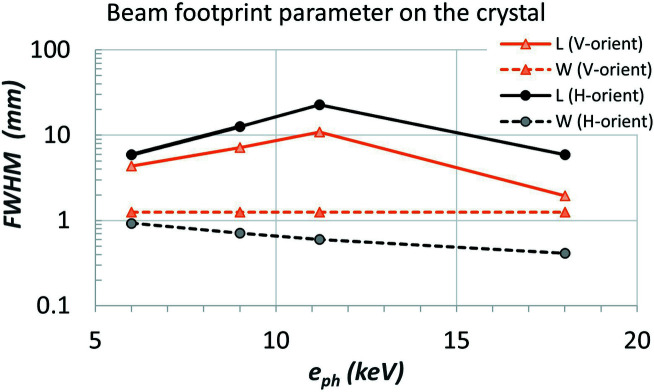
Beam footprint parameters FWHM for both vertical and horizontal orientated crystal for the four cases listed in Table 1 ▸. The effective beam footprint size is two times the FWHM. L – beam footprint length in the meridional direction; W – beam footprint width in the sagittal direction.

The minimized residual height error std_d*Uy*_ in the (beam) horizontal direction is plotted in Fig. 7 ▸ for both cases when the crystal is in a vertical or horizontal orientation. These results clearly show that the thermal deformation of the vertically oriented crystal is smaller than that of the horizontally oriented crystal. This difference can be explained by the beam footprint or power distribution. The photon beam size is a constant 2×FWHM = 2.5 mm in the horizontal direction and decreases from 2×FWHM = 2.08 to 0.88 mm when the photon energy varies from 6 to 18 keV. The total power and peak power density are identical for both crystal orientations. But the footprint shape is different: a more elongated elliptical shape for the horizontal orientation than for the vertical orientation (see Fig. 8 ▸ for the beam footprint size). With the same total power and peak power density, the longer the footprint length, the higher the thermal deformation in the meridional direction.

**Figure 9:**
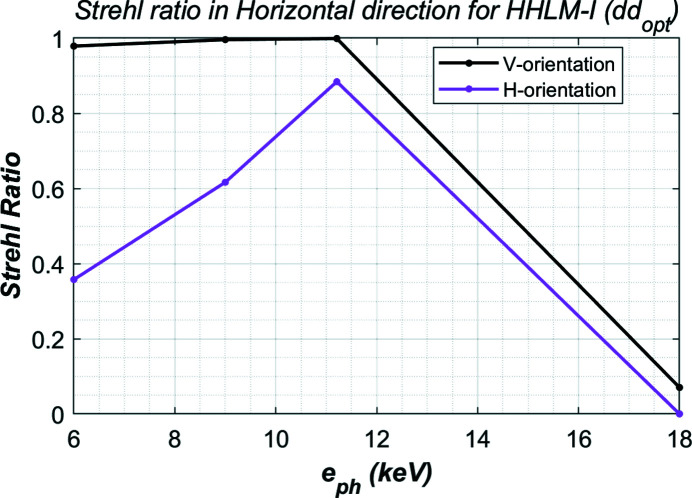
Strehl ratio of the crystal for both vertical and horizontal orientated crystals for the four cases (photon energies) listed in Table 1 ▸ and 70 W absorbed power.

From the height error shown in Fig. 7 ▸, one can calculate the Strehl ratio of the crystal using equation (7). Results are plotted in Fig. 9 ▸. Consistent with thermal deformation of the crystal shown in Fig. 8 ▸, the Strehl ratio of the vertically oriented crystal is nearly equal to 1 up to 12 keV, and higher than the horizontally oriented crystal. The Strehl ratio at 18 keV is very small. This is because the higher-order reflection crystal Si(333) is used at 18 keV. The beam footprint at 18 keV is smallest, the power density is highest. Therefore, the thermal deformation at 18 keV is highest. In practice, the FEL power from LCLS-II(-HE) decreases when the photon energy increases. By reducing the total FEL power at 18 keV from 100 W to 70 W (or absorbed power from 70 W to 50 W), a Strehl ratio of 0.9 can be achieved at 18 keV.

## 4. Wavefront propagation simulation

To confirm the expectations based on the Strehl ratios calculated in Section 3, we also performed one-dimensional wave-optical simulations to investigate the focusing properties of the hard X-ray beam upon reflection from the distorted crystal surfaces. In addition, we explore methods for mitigating the majority of the negative effects via adjustment of a bendable mirror located a few metres upstream of the crystal.

### 4.1. Methodology of the wavefront propagation simulation

The wavefront propagation method used here is inspired by that employed by the *Synchrotron Radiation Workshop* (*SRW*) software (Chubar & Celestre, 2019 ▸). The method relies heavily on the Fresnel scaling theorem for efficient computation of wavefront propagation between optics, keeping track of the quadratic part of the wavefront analytically and thus avoiding the need to properly sample strong phase curvature (Paganin, 2006 ▸). The wavefront interaction with optics is taken care of using a hybrid ray-tracing wave-optics approach, using knowledge of the wavefront curvature to compute the ‘local’ angles of incidence and subsequent reflection or diffraction at every point on a given optic; this is important for dispersive reflections such as in the case of asymmetric crystals, as well as for computing complex-valued crystal reflectivities based on the incident angle. While the crystal deformations considered here have slope errors much smaller than the Darwin width of Si(111) at 10 keV, it is worth noting that the crystal reflectivities are computed using functions from the back-end for the *XRayTracer* (*xrt*) ray-tracing package (Klementiev & Chernikov, 2014 ▸). Finally, the impact on the wavefront of nanometre (and picometre) scale deformations is computed by numerical integration of the slope error of the resulting reflection. The codes used for the simulations are freely available on the web (https://github.com/mseaberg/lcls_beamline_toolbox).

### 4.2. Results of intensity distribution

**Figure 10:**
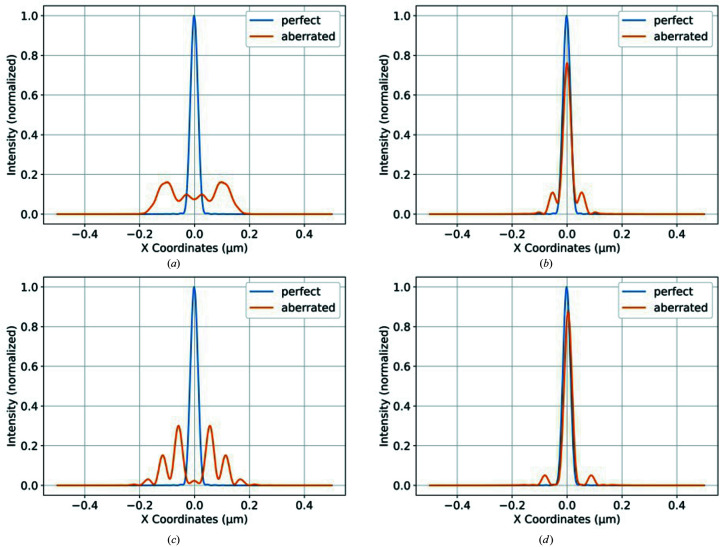
Normalized intensity distributions at the focus location without thermal deformation (perfect) or with thermal deformation (aberrated) for all four cases listed in Table 2 ▸. (*a*) *P*_abs_ = 5 W, *T*_cool_ = 80 K, std_*Uy*_ = 170 pm, SR ≃ 0. (*b*) *P*_abs_ = 5 W, *T*_cool_ = 80 K, std_d*Uy*_ = 34 pm, SR = 0.62. (*c*) *P*_abs_ = 50 W, *T*_cool_ = 115.5 K, std_*Uy*_ = 444 pm, SR ≃ 0. (*d*) *P*_abs_ = 50 W, *T*_cool_ = 112.5 K, std_d*Uy*_ = 24 pm, SR = 0.79.

To illustrate the consequences of the thermal deformation of the crystal on the beam intensity distribution, we have focused on the crystal C_1-1_–Si(111) with three degrees asymmetry cut and photon energy at 10 keV. Four cases [(*a*)–(*d*); Table 2 ▸] have been considered in the horizontal orientation. The wavefront propagation was simulated through the periscope mirrors and the first crystal in the HHLM. To gauge the effect of thermal deformations on focusing quality the resulting wavefront was propagated through an ideal lens with 1 m focal length. The intensity distributions at the focus location without thermal deformation (perfect) or with thermal deformation (aberrated) are compared in Fig. 10 ▸ for all four cases (*a*)–(*d*) listed in Table 2 ▸.

With 5 W absorbed power and standard LN2 cooling, the height error of the crystal std_*Uy*_ is 170 pm, and the Strehl ratio is close to 0. The focus becomes blurred: beam intensity at the centre of the beam reduced by a factor of ten, and the beam size is increased by a factor of about six compared with the perfect crystal [Fig. 10 ▸(*a*)]. Using a bendable focusing mirror (periscope mirror) to compensate the second-order component in the thermal deformation, the residual height error std_d*Uy*_ can be reduced to 34 pm, and the Strehl ratio reaches 0.62. The beam profile and intensity [Fig. 10 ▸(*b*)] are significantly improved compared with the uncompensated case [Fig. 10 ▸(*a*)].

With 50 W absorbed power and optimized cooling temperature, the uncompensated height error std_*Uy*_ [case (*c*)] reaches 444 pm, and the Strehl ratio is close to 0. The beam aberration is greater than for case (*a*) with the beam intensity at the centre of the beam reduced to nearly 0, and the beam profile is uglier. The compensation by the focusing mirror of the second-order component in the thermal deformation allows the residual height error std_d*Uy*_ to be reduced to 24 pm, and the Strehl ratio is increased to 0.79. The reflected beam intensity profile is satisfactory: peak intensity reduction of about 20% and a very small tail of less than 5% of peak intensity. Note that when optimizing the temperature based on the compensated height error (std_d*Uy*_) versus uncompensated height error (std_*Uy*_) there is a 3 K difference.

The ratio of the peak intensity with aberrated crystal to the perfect crystal in the cases (*b*) and (*d*) corresponds well to the value of the Strehl ratio estimations calculated with equation (7): 0.62 for case (*b*) and 0.79 for case (*d*).

Considering the relevance of the thermal deformation requirement and the Strehl ratio, the ratio of the peak intensity of the reflected beam by the crystal with aberration related to the perfect crystal is close to the Strehl ratio when the ratio of the intensities is larger than the 10%. When the Strehl ratio is small (<0.1), the ratio of the peak intensity is no longer relevant. The Strehl ratio is calculated with a global metric of thermal deformation – the RMS height error Δ*h*. However, the local thermal deformation (slope or height error) over the footprint is variable; there is always a small region around the centre of the beam footprint where the local thermal deformation is very small.

## 5. Summary and conclusion

In this paper, we revisited the state-of-the-art solutions to minimize the thermal deformation of X-ray optics, detailed the optical requirements for wavefront preservation, and formulated the criteria on thermal deformation of X-ray optics, especially for crystal monochromators. These criteria can be used to guide opto-mechanical engineering design decisions. The height error standard deviation requirement is sub-nm for mirrors (surface shape), and smaller than 25 pm for crystal monochromators (crystal plane). Such unprecedented requirements are pushing opto-mechanical engineering efforts to address this challenge. We have demonstrated that the thermal deformation of a crystal monochromator cooled by liquid nitro­gen can be minimized by optimizing the effective cooling temperature. A factor of ten in performance can be achieved compared with standard liquid-nitro­gen cooling. Using dynamic focusing elements (for instance a focusing mirror) to compensate the second-order component of the thermal deformation, we can reduce the residual height error by another order of magnitude. As an example, for the LCLS-II-HE DXS instrument the criteria on thermal deformation of the HHLM crystal can be achieved for a 100 W SASE FEL power load. The wavefront propagation simulations confirm that the reflected beam intensity profile is satisfactory both in the peak power density and focused beam size.

The path forward includes the detailed engineering design to implement the proposed solution for crystal monochromators: optimization of effective cooling temperature, compensation of the second-order component in the thermal deformation, and minimization of the fabrication/mounting/cooling-induced deformation.

## Figures and Tables

**Table 1:** Optimal effective cooling temperature *T*_cool-opt_ (K) for minimized residual height error std_d*Uy*_, and the maximum temperature in the crystal *T*_max_ (K)

Case number	Crystal, photon energy	*T*_cool-opt_ (K)	*T*_max_ (K)
1	Si(111), 6 keV	100	128.2
2	Si(111), 9 keV	104	127.0
3	Si(111), 11.21 keV	107	126.4
4	Si(333), 18 keV	92.5	131.7

**Table 2:** Parameters of the four cases (*a*, *b*) Fixed effective cooling temperature *T*_cool_. Optimized effective cooling temperature *T*_cool_ for minimized (*c*) height error std_*Uy*_, (*d*) residual height error std_d*Uy*_; second-order correction using bendable periscope mirror.

Case	Absorbed power *P*_abs_ (W)	Second-order (best fit)	Effective cooling temperature
(*a*)	5	Not corrected	80 K
(*b*)	5	Corrected	80 K
(*c*)	50	Not corrected	115.5 K optimized for std_*Uy*_
(*d*)	50	Corrected	112.5 K optimized for std_d*Uy*_
